# A Treatise on Gynecology—Medical and Surgical

**Published:** 1892-09

**Authors:** 


### Book Reviews.

A TREATISE ON GYNECOLOGY, MEDICAL AND SURGICAL.—By S. Pozzi, M. D., Adjunct Professor at the Faculty of Medicine, etc., Paris. Translated from the French edition under the supervision of, and with additions by, Brook H. Wells, M. D., Lecturer on Gynecology, New York Polyclinic, etc. Published in two volumes. Published by Wm. Wood & Co., New York. Sold by subscription.

VOL. II. contains 583 pages, 174 wood cuts and 9 full page colored plates. It treats of inflammations, cysts and neoplasms of uterine appendages and ligaments, genital tuberculosis, intra- and extra-peritoneal pelvic hematocele, extra-uterine pregnancy, diseases and injuries to vagina and vulva, malformation of the genital organs, with an addition by translator of two chapters on diseases of urinary tract, rectum and pelvis, taken from Auvard's "Traite Pratique de Gynecologie." Under oophoro-salpingitis, the author states that "he does not believe that true ovaritis exists without previous endometritis or salpingitis, that it is a mixed lesion; that inflammatory conditions of uterus are, without doubt, the chief cause of inflammations of the appendages," p. 7. Under cystic oophoro-salpingitis, pyo-hydro- and hemato-salpinx, are duly considered.

In treatment of acute salpingitis, he advises dilatation of cervix, curetting and iodine applications to endometrium. Does not consider electricity of much utility in diseases of appendages.

Forty pages are devoted to pathological anatomy of ovarian cysts, with one page to cysts of corpus luteum. Etiology, diagnosis, prognosis and treatment of ovarian cysts occupies 52 pages.

Chapter VIII. deals with solid tumors of ovaries.

Chapter IX. deals with tumors of Fallopian tubes, round and broad ligaments.

Chapter X. is devoted to tuberculosis, genital and peritoneal.

Chapter XI. considers intra- and extra-peritoneal pelvic hematocele.

Page 223, we find: "The effusion of blood into the pelvic cavity is probably quite frequent; there is no doubt that at the menstrual period the tubes are the seat of an exudation of blood as well as the uterus. Many menstrual disorders caused by fatigue, strain or cold, are really due to the effusion of a small amount of blood into peritoneal cavity, where it is rapidly absorbed." He also states that "hematocele is bi-lateral, that tubal pregnancy is not to be thought of, nor can we admit a reflux of blood from the abdomen into the tubes. The usual origin of extensive effusions of blood is undoubtedly the early rupture of an extra-uterine tubal pregnancy."

C.apter XII. is devoted to extra-uterine pregnancy.

Diseases and injuries of the vagina and vulva with malformation of the female genitalia, occupy a considerable part of the work. Diseases of the urinary tract, rectum and pelvis are disposed of in two chapters, which are translated from Auvard's recent work.

The two volumes are a valuable contribution to the gynecological literature of the day. The translator has done his work well, while the plates and binding are well executed.

